# Impact of chemokine C–C ligand 27, foreskin anatomy and sexually transmitted infections on HIV-1 target cell availability in adolescent South African males

**DOI:** 10.1038/s41385-019-0209-6

**Published:** 2019-10-16

**Authors:** Clive M. Gray, Kyle L. O’Hagan, Ramon Lorenzo-Redondo, Abraham J. Olivier, Sylvie Amu, Nyaradzo Chigorimbo-Murefu, Rushil Harryparsad, Shorok Sebaa, Lungile Maziya, Janan Dietrich, Kennedy Otwombe, Neil Martinson, Selena Ferrian, Nonhlanhla N. Mkhize, David A. Lewis, Dirk Lang, Ann M. Carias, Heather B. Jaspan, Douglas P. K. Wilson, Marcus McGilvray, Gianguido C. Cianci, Meegan R. Anderson, Minh H. Dinh, Anna-Lise Williamson, Jo-Ann S. Passmore, Francesca Chiodi, Thomas J. Hope

**Affiliations:** 1 Division of Immunology, Institute of Infectious Disease and Molecular Medicine, Department of Pathology, University of Cape Town, Cape Town, South Africa; 2 National Health Laboratory Service, Cape Town, South Africa; 3 Division of Infectious Diseases, Northwestern University Feinberg School of Medicine, Chicago, IL 60011 USA; 4 Division of Virology, Institute of Infectious Diseases and Molecular Medicine, Department of Pathology, University of Cape Town, Cape Town, South Africa; 5 Department of Microbiology, Tumor and Cell Biology at Biomedicum, Karolinska Institutet, Stockholm, Sweden; 6 Department of Internal Medicine, Edendale Hospital, Pietermaritzburg, South Africa; 7 Perinatal HIV Research Unit, SAMRC Soweto Matlosana Collaborating Centre for HIV/AIDS and TB, Faculty of Health Sciences, University of the Witwatersrand, Johannesburg, South Africa; 8 National Institute for Communicable Diseases, Sandringham, Johannesburg, South Africa; 9 Westmead Clinical School, Faculty of Medicine and Health & Marie Bashir Institute for Infectious Diseases and Biosecurity, University of Sydney, Sydney, Australia; 10 Department of Human Biology, University of Cape Town, Cape Town, South Africa; 11 Department of Cell and Developmental Biology, Northwestern University Feinberg School of Medicine, Chicago, IL USA; 12 Seattle Children’s Research Institute and University of Washington Departments of Pediatrics and Global Health, Seattle, WA USA; 13 Whizzkids United, Edendale, Pietermaritzburg, South Africa

## Abstract

We compared outer and inner foreskin tissue from adolescent males undergoing medical male circumcision to better understand signals that increase HIV target cell availability in the foreskin. We measured chemokine gene expression and the impact of sexually transmitted infections (STIs) on the density and location of T and Langerhans cells. Chemokine C–C ligand 27 (CCL27) was expressed 6.94-fold higher in the inner foreskin when compared with the outer foreskin. We show that the density of CD4^+^CCR5^+^ cells/mm^2^ was higher in the epithelium of the inner foreskin, regardless of STI status, in parallel with higher CCL27 gene expression. In the presence of STIs, there were higher numbers of CD4^+^CCR5^+^ cells/mm^2^ cells in the sub-stratum of the outer and inner foreskin with concurrently higher number of CD207^+^ Langerhans cells (LC) in both tissues, with the latter cells being closer to the keratin surface of the outer FS in the presence of an STI. When we tested the ability of exogenous CCL27 to induce T-cell migration in foreskin tissue, CD4 + T cells were able to relocate to the inner foreskin epithelium in response. We provide novel insight into the impact CCL27 and STIs on immune and HIV-1 target cell changes in the foreskin.

## Introduction

Three randomised controlled trials (RCTs) in eastern and South Africa demonstrated that Medical Male Circumcision (MMC) provided 52–64% protection from HIV infection^1–3^ and spurred the roll-out of voluntary MMC as a prevention measure throughout South Africa.^4^ However, only 1.9 million of the targeted 4.3 million MMCs have been performed, demonstrating difficulty in uptake of this procedure. Although it is clear that MMC reduces HIV infection in men, the mechanism of circumcision-induced reduction of HIV acquisition is not clear. Understanding mechanisms of MMC-mediated protection against HIV may allow development of alternative options for prevention.

Several theories have been posited to explain the protective mechanism of MMC.^5^ One is that the inner foreskin is less keratinised than the outer foreskin, having physiological characteristics more similar to mucosal columnar epithelia than the squamous epithelial morphology of the outer foreskin and penile shaft.^6,7^ Early qualitative studies seemed to support the hypothesis that the inner foreskin was less keratinised than the outer foreskin,^8–10^ which may result in easier access to HIV target cells. However, subsequent studies that measured and statistically analysed the thickness of the superficial keratin layer found no keratin thickness difference between the inner and outer foreskin.^11–13^ Other possible mechanisms of protection include the removal of HIV target cells following circumcision. The foreskin has been shown to harbour high densities of HIV target cells, most notably CD4 + T cells and Langerhans cells.^14,15^ Target cells have been identified in both the inner and outer foreskin, to varying degrees.^11,16,17^ Comprehensive phenotyping has shown that foreskin T cells are skewed towards either a resting or effector memory phenotype, expressing increased levels of the HIV co-receptor, CCR5, and producing higher levels of pro-inflammatory IL-17 relative to circulating T cells in the blood.^15,18^ These studies therefore suggest that the foreskin has a resident population of active immunecompetent cells. It may thus not be surprising that men with a larger foreskin surface area were shown to be at higher risk of HIV acquisition.^19^ Consequently, the protective benefits of MMC may be a result of removal of tissue that possesses a significant proportion of HIV target cells that are primed for infection. There is currently limited evidence for any of these possible mechanisms of protection following MMC.

Sexually transmitted infections (STIs) represent a significant HIV risk factor, increasing risk twofold to threefold in uncircumcised males.^5,20^ As an example, asymptomatic HSV-2 infection has been implicated in increasing HIV target cells in the foreskin and compromising skin barrier integrity by reducing expression of the tight junction protein, Claudin.^21^ This suggests that other asymptomatic STIs may also contribute to increased HIV acquisition risk by potentially inducing changes in immune cell populations along with compromised skin barrier integrity.

In this study, we recruited adolescent males in South Africa who were undergoing elective MMC with the aim of understanding the impact of chemokines and STIs on inducing the availability of HIV target cells in the foreskin. When we compared the outer and inner foreskins for chemokine gene expression profiles, chemokine C–C ligand 27 (CCL27) was significantly elevated in the inner foreskin relative to the outer foreskin, regardless of STI status. We show there were significantly higher numbers of HIV target cells in both outer and inner foreskins from adolescents with a detectable asymptomatic STI, notably *Chlamydia trachomatis*.

## Results

### Selection of participants with an asymptomatic STI

Up to 42 adolescent males were included in the study, 21 infected with a detectable STI in first pass urine and broken down as follows: *Neisseria gonorrheae* (NG, *n* = 1), *Chlamydia trachomatis* (CT, *n* =  10) or with CT coinfected with NG, MG and MG/NG (*n* = 4), *Trichomonas vaginalis* (TV, *n* = 2), *Mycoplasma genitalium* (MG, *n* = 3), HSV-1 and HSV-2 (*n* = 1). We then selected foreskin tissue from age-matched STI-negative participants as controls to compare with the STI-positive participants (up to 21). A total of 150 males were screened for STIs, and the prevalence of any STI was 14% with CT being the most prevalent (*n* = 14; 67%). All infections were asymptomatic.

### Chemokine C–C ligand 27 (CCL27) gene expression distinguishes the inner from the outer foreskin

**Fig. 1:**
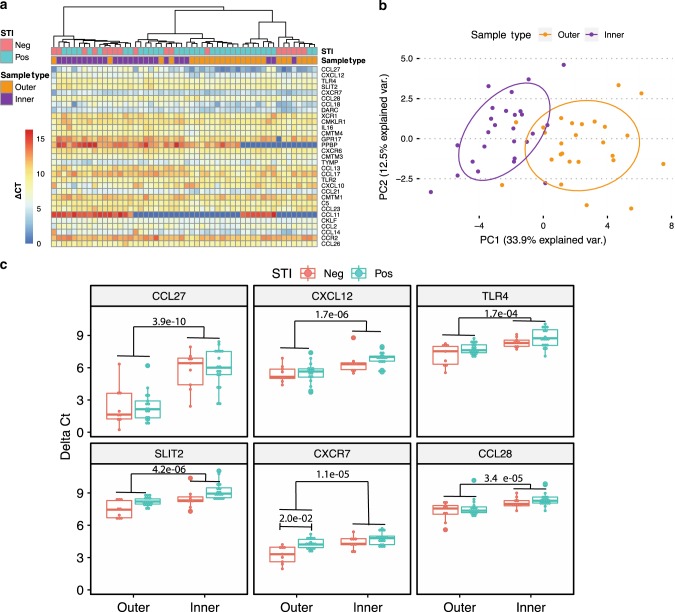
Chemokine gene expression profiles in the foreskin tissue after MMC from STI_neg_ and STI_pos_ participants. **a** Heatmap of the ΔCT values of the significantly differentially expressed genes between the inner and outer foreskin. Hierarchical clustering was used to cluster the samples analysed. **b** Principal component analysis of gene expression of the significant genes. A clear clustering of the samples between the inner and outer foreskin can be observed driven mainly by PC1, which reflects the difference of expression levels of the significant genes between these two tissue types. **c** Boxplot of the six most significant differentially expressed genes. The data are grouped by tissue type (*x*-axis) and STI infection (colour). The *q*-values are shown for the significant contrasts (FDR < 0.05), including only the significant genes between STIpos and STIneg. Tukey’s box and whisker plots were used and box limits: interquartile range (IQR); middle line: median; vertical lines: adjacent values (1st quartile − 1.5 IQR; 3rd quartile + 1.5 IQR)

We assessed the expression of an array of chemokine genes in the outer and inner foreskin tissue and whether the presence of an STI had an effect on this expression. A targeted microarray approach was used to measure 84 chemokine genes in dissected outer and inner foreskin tissue from participants positive or negative for STIs. By comparing the outer and inner foreskin, we aimed to show that these tissue compartments are different. We built a linear model with the normalised expression levels of every gene to estimate the effects of the outer and inner foreskin, STI infection and the combination of these two factors, controlling for within participant correlation. We excluded from the analysis the data from two participants who showed significantly lower average gene expression values. By testing for differential expression between all the possible contrasts within the model, we found that the majority of significant genes expression differences were between the outer and inner foreskin. Of the 84 genes examined, 31 (37%) differed significantly between the outer and inner foreskin using a false discovery rate (FDR) of 0.05. Hierarchical clustering and principal components analysis (PCA) of the variation in the mRNA levels of the significant genes highlighted a clear clustering of the samples between the outer and inner foreskin (Fig. 1a, b). From this analysis, the following 28 genes were upregulated in the inner foreskin compared with outer: CCL27, CXCL12, TLR4, SLIT2, CXCR7, CCL28, CCL18, DARC, XCR1, CMKLR1, IL16, CMTM4, GPR17, PPBP, CXCR6, CMTM3, CCL13, CCL17, CCL21, CMTM1, C5, CCL23, CCL11, CKLF, CCL2, CCL14, CCR2 and CCL26 (Fig. 1a; Supplementary Table 1). Conversely, there was an upregulation of CXCL10, TLR2 and TYMP in the outer foreskin relative to the inner tissue. Half of these genes (15) were also significantly differentially expressed between the outer and inner foreskin when considering the STI_neg_ participants only (Supplementary Table 1). Among the significant genes between the outer and inner foreskin, CCL27 stood out with a 6.94-fold (*q* = 3.9 × 10^−10^) increase in the inner foreskin (Fig. 1c). Only CXCR7 gene expression was differentially upregulated in presence of an STI, with a 1.74-fold increase (*q* = 2 × 10^−2^) in the outer foreskin (Fig. 1c; Supplementary Table 1).

Collectively, our data show that CCL27 was the most highly elevated expressed gene in the inner foreskin when compared with the outer foreskin, and there was increased CXCR7 gene expression in the outer foreskin in the presence of an STI.

### Influence of CCL27 on the density and location of CD4^+^CCR5^+^ cells in the inner and outer foreskin

**Fig. 2:**
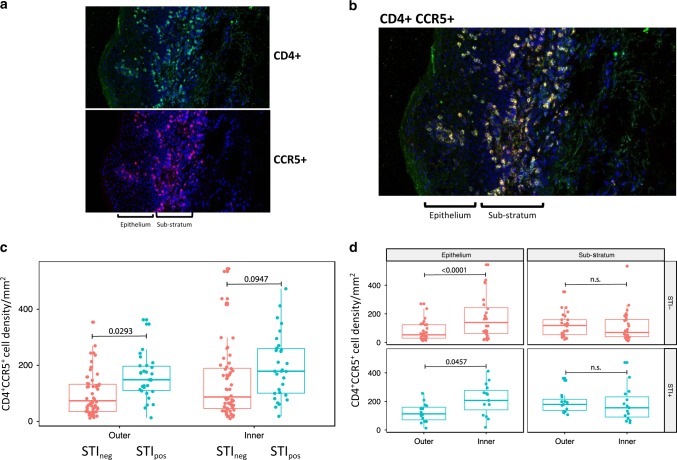
Impact of asymptomatic STIs on the density of CD4^+^CCR5^+^ T cells in the foreskin tissue after MMC from STI_neg_ and STI_pos_ participants. **a** Representative stained images of FS for CD4 (green) and DAPI (blue), and CCR5 (red) and DAPI (blue), with the epithelium and sub-stratum indicated. **b** Representative triple-stained image of an inner foreskin (FS) tissue for CD4 (green) and CCR5 (red) and DAPI (blue). **c** Comparison of CD4 + CCR5 + cell density in the epithelium and sub-stratum (per mm^2^) in matching inner and outer foreskin tissue between STI_neg_ and STI_pos_ participants (*n* = 28 for matching inner and outer FS from STI_neg_, *n* = 17 for matching inner and outer FS from STI_pos_). A negative binomial mixed-effects model was used, and the horizontal line is the median value and lower and upper error bars are the interquartile ranges. **d** Comparison of CD4 + CCR5 + cell numbers (double positive cells per mm^2^ of tissue) in matching inner and outer foreskin tissue between STIneg and STIpos participants. A total of five images per inner and outer FS from STIneg and STIpos males were averaged (*n* = 28 for matching inner and outer FS from STIneg, *n* = 17 for matching inner and outer FS from STIpos). A negative binomial mixed-effects model was used, and the horizontal line is the median value and lower and upper error bars are the interquartile ranges

CCL27 is a chemokine that is associated with the recruitment of memory T cells to the skin and interacts with CCR10 on T cells to mediate recruitment and function.^22,23^ As the CCL27 gene signal was more highly expressed in the inner foreskin, we hypothesised that this would result in an increased density of CD4^+^CCR5^+^ cells in the epithelium of the inner foreskin. Therefore, we examined the density of CD4 + CCR5 + cells in the outer and inner foreskin of our cohort and stained cryosectioned tissue for CD4 and CCR5 using fluorescent secondary antibodies. Representative images are shown in Fig. 2a, b highlighting the very high occurrence of double-stained CD4 + CCR5 + cells in the tissue. Figure 2c shows that males infected with an STI had a significantly higher density of CD4^+^CCR5^+^ cells in the outer foreskin (*q* = 0.0293) when combining the epithelium and sub-stratum cell counts. A similar trend was also observed in the inner foreskin (Fig. 2c). The median number of CD4^+^CCR5^+^ cells/mm^2^ was 77 cells/mm^2^ in STI_neg_ outer foreskin and 121 cells/mm^2^ STI_pos_ tissue. For the inner foreskin, those densities were 171 cells/mm^2^ for STI_neg_ versus 211 cells/mm^2^ for STI_pos_ samples. The higher numbers of CD4^+^CCR5^+^ cells/mm^2^ in foreskins from STI_pos_ participants appeared to be driven by higher numbers of these cells in the sub-stratum, and not in the epithelium (Supplementary Fig. 1). Noteworthy from our analysis was that the density of CD4^+^CCR5^+^ cells was significantly higher in the epithelium of the inner foreskin when compared with the epithelium of the outer foreskin (Fig. 2d) for both STI_neg_ (75 cells/mm^2^ vs 174 cells/mm^2^; *q* < 0.0001) and STI_pos_ groups (117 cells/mm^2^ vs 212 cells/mm^2^; *q* = 0.0457). The density of CD4^+^CCR5^+^ cells in the sub-stratum of the inner and outer foreskin tissue was not significantly different for either STI_neg_ (109 cells/mm^2^ vs 120 cells/mm^2^) or STI_pos_ (185 cells/mm^2^ vs 206 cells/mm^2^).

**Fig. 3:**
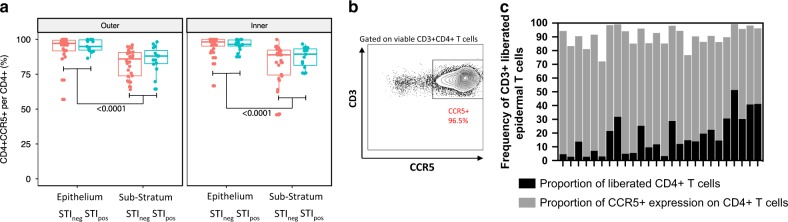
CCR5 co-expression on CD 4+ cells in tissue and in migrated cells from the epidermis. **a** Comparison of %CCR5 expression on CD4 + T cells in matching inner and outer foreskin tissue between STI_neg_ and STI_pos_ participants (*n* = 28 for matching inner and outer FS from STI_neg_, *n* = 17 for matching inner and outer FS from STI_pos_). The horizontal line is the median value, and lower and upper error bars are the interquartile ranges. **b** A representative flow-cytometric contour plot (with outliers) showing expression of CCR5 on migrated CD3^+^CD4^+^ T foreskin tissue cells. **c** Proportion of CCR5 expression (shaded bars) on epidermal CD3^+^CD4^+^ T cells (solid bars) from 13 fresh foreskins

Our data also showed higher CCR5 co-expression on CD4 + cells in the epithelium of both outer (median of 97%) and inner tissue (median of 98%) compared with the sub-stratum outer (median of 86%) and inner (89%) tissue (Fig. 3a; *q* < 0.0001), and appeared to be unrelated to STI status. Confirmation of high co-expression of CCR5 on CD3^+^CD4^+^ cells in the epithelium was made using flow cytometry, where T cells were allowed to spontaneously migrate out of epidermal sheets from the inner foreskin. Figure 3b shows a representative flow cytometry plot of CCR5 co-expression and was reproduced in 13 different donors (Fig. 3c), where despite foreskin tissue having variable proportions of CD4 + T cells (mean = 18 ± 13% of CD3 + cells), there was an average CCR5 co-expression of 90 ± 7% on CD4 + T cells. Collectively, our data show that the numbers of CD4^+^CCR5^+^ cells/mm^2^ are higher in the epithelium of the inner foreskin, and that CCR5 co-expression on CD4 + cells was highest in the epithelium of both the outer and inner foreskin. The presence of an STI resulted in a higher density of CD4^+^CCR5^+^ cells/mm^2^ appearing in the sub-stratum of both the outer and inner foreskin. This coincides with the almost sevenfold higher expression of CCL27 in the inner foreskin.

### Impact of STIs on keratin thickness, Langerhans Cell density and spatial organisation

**Fig. 4:**
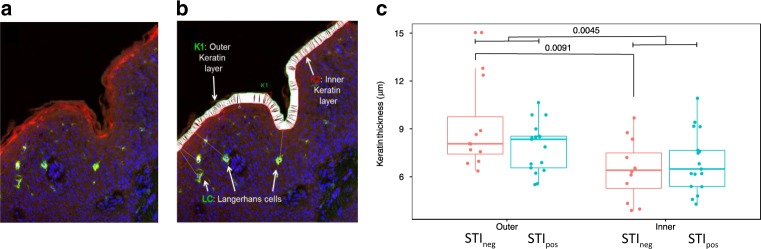
Keratin thickness in foreskins from STI_neg_ and STI_pos_ participants. **a** Representative stained images of the foreskin (FS) for fillagrin (red), CD207 (green) and DAPI (blue). **b** Fillagrin thickness was determined using Integrative Data Language (IDL) after demarcating the outer keratin layer (K1) and the inner keratin layer (K2) and used to study STI_neg_ and STI_pos_ participants (including both matching inner and outer foreskin). **c** Comparison of keratin thickness between STI_neg_ and STI_pos_ participants. A total of five images per inner and outer FS from age-matched STI_neg_ and STI_pos_ males was averaged (*n* = 12 for STI_neg_, *n* = 17 for STI_pos_)

To assess whether STIs had an influence on epithelial keratinisation, inner and outer foreskin tissue was stained for filaggrin (a marker of keratin). The position of Langerhans cells (LC) within inner and outer foreskin and filaggrin staining thickness (distance demarcated between K1 and K2) was measured using Integrative Data Language (Fig. 4a, b). Participants were stratified by outer and inner foreskin, and filaggrin thickness measured for both STI_neg_ and STI_pos_ groups (Fig. 4c). The median filaggrin thickness significantly differed between the outer and inner foreskins of young men who were either STI_neg_ or STI_pos_ (Fig. 4c; *q* = 0.0045), with no difference between STI status. This difference was more pronounced in males who were STI_neg_, where there was a significant (*q* < 0.0091) mean thickness difference of 2.67 µm between the inner and outer foreskin, showing that the outer foreskin was more keratinised. STIs had no impact on epithelial keratinisation

**Fig. 5:**
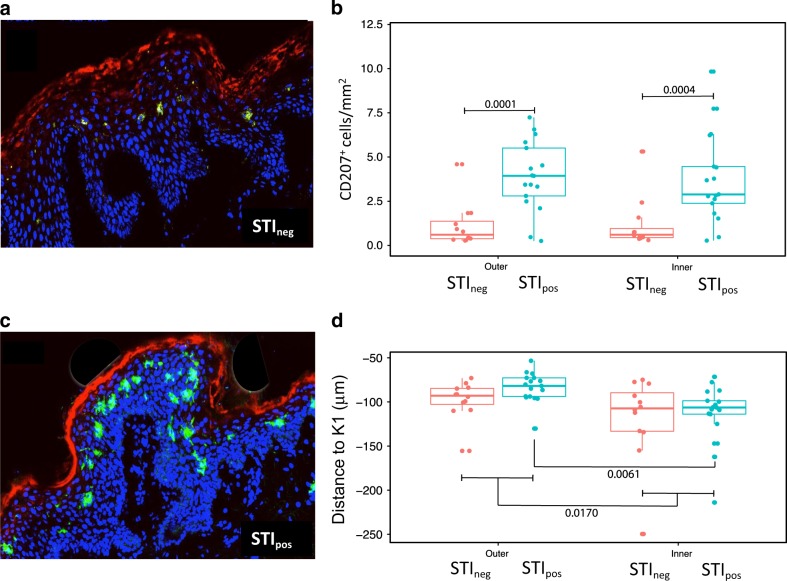
Density and spatial distribution of Langerhans cells from STI_neg_ and STI_pos_ participants. **a**, **b** Confocal images for fillagrin (red), CD207 (green) and DAPI (blue) from the inner foreskin of a representative STI_neg_ (**a**) and STI_pos_ (**b**) participant with CT infection. **c** Comparison of CD207 + cells per mm^2^ of tissue from matching inner and outer foreskin tissue between STI_neg_ and STI_pos_ participants. A total of five images per inner and outer FS from age-matched STI_neg_ and STI_pos_ males were averaged (*n* = 12 for STI_neg_, *n* = 17 for STI_pos_). **d** Comparison of CD207 + cell distance from the outer keratin layer (K1) in matching inner and outer FS between STI_neg_ and STI_pos_ participants. A total of five images per inner and outer FS from age-matched STI_neg_ and STI_pos_ males were averaged (*n* = 12 for STI_neg_, *n* = 17 for STI_pos_). A log-normal model was used to test the significance in foreskin measurements between STI_neg_ and STI_pos_ males. In all plots, the horizontal line is the median value and lower and upper error bars are the interquartile ranges

To determine whether the presence of STIs impacted the numbers and spatial arrangement of LCs, a known HIV target cell,^24^ we stained foreskin tissue with CD207. Figure 5a, b shows CD207 staining in representative images within inner foreskins taken from two participants who were either STI_neg_ (Fig. 5a) or STI_pos_ (Fig. 5b). A representative isotype stained control is shown in Supplementary Fig. 3. Figure 5c shows significantly higher densities of CD207^+^ in both the outer and inner foreskins from participants with any STI. Except for *Chlamydia trachomatis* (CT), there were too few other STIs detected to identify whether there was preferential elevation of LC with different STIs. In addition to the density of LC, we measured the distance of cells within the epithelial tissue to the outer aspect of the keratin layer (K1 in Fig. 5b). Figure 5d shows that overall LCs were closer to the keratin surface in the outer foreskin (*q* = 0.017), which appeared to be due to the impact of STI, where LCs found in the outer foreskin from participants who were STI_pos_, were spatially arranged much closer to the keratin surface when compared with inner foreskin (Fig. 5d; *q* = 0.0061).

Overall, these data show that CD207^+^ LCs are more abundant in the presence of asymptomatic STIs and that they lie closer to the keratin layer in the outer foreskin.

### Exogenously added CCL27 protein promotes migration of tissue-resident T cells

**Fig. 6:**
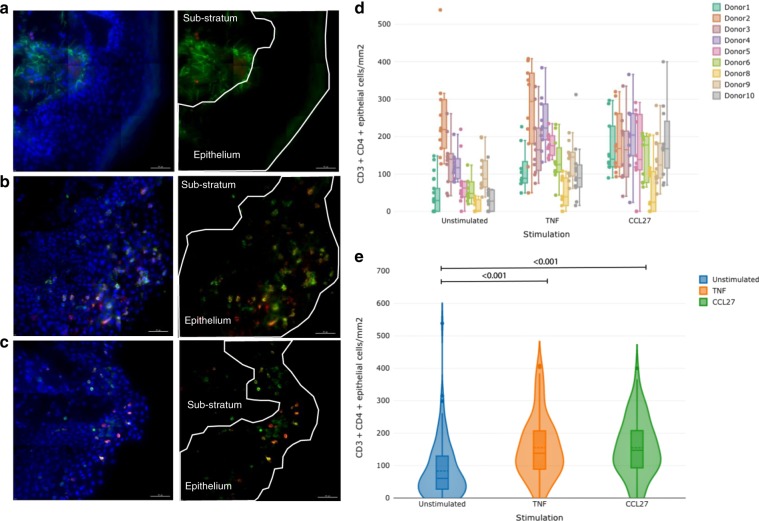
Effect of CCL27 on CD3^+^CD4^+^ T-cell migration into the inner foreskin epithelium. **a**–**c** Inner foreskin tissue cultured with media alone (unstimulated); **a**, TNF-α 100 ng/mL; **b** or CCL27 400 ng/mL; **c** for 48 h. Stimulated tissue was snap-frozen, sectioned and stained with antibodies against human CD3 (green), human CD4 (red) and counterstained with DAPI (blue). Merged images (left) and merged images without DAPI (right) are shown. All images were captured with a ×60 objective. The white polygon (right panel) illustrates the surface area of the epithelium in each sample, with the sub-stratum and epithelium indicated. Scale bars represent 40 µm. **d** A negative binomial generalised linear mixed-effects model was used to test the differences in cell counts, controlling for within donor correlation and using the area analysed per image as an offset of the model. Tukey’s box and whisker plots; box limits: interquartile range (IQR); middle line: median; vertical lines: adjacent values (1st quartile − 1.5 IQR; 3rd quartile + 1.5 IQR). **e** Violin plot and boxplot showing the effect of TNF-α and CCL27 stimulation on the density of CD3^+^CD4^+^ epithelial cells in the inner foreskin from nine different donors. The data were grouped by the stimulation used. The statistically significant comparisons are shown

Knowing that CCL27 gene expression was highly differentially expressed in the inner foreskin and that CD4 + CCR5 + cell density was higher in the epithelium of inner foreskin than in outer foreskin, we wished to test the hypothesis that CCL27 protein could cause migration of tissue-resident CD4^+^ T cells into the epithelium of the inner foreskin. Figure 6a–c shows representative fluorescent-stained images depicting the presence of CD3^+^CD4^+^ dually stained cells appearing in the epithelium before and after culture of STI_neg_ inner foreskin tissue with TNF (positive control) and CCL27. Figure 6d shows the individual variation of the ten foreskin donors used to assess cell migration, where most individual tissue cultures showed an increase in CD4 + T cells in epithelium. The collated data (Fig. 6e) show a highly significant twofold to threefold (*q* < 0.001) increase in CD3 + CD4 + T cell numbers in the epithelium in response to both TNF (from 60 cells/mm^2^ to 138 cells/mm^2^) and CCL27 (from 60 cells/mm^2^ to 147 cells/mm^2^). Supplementary Fig. 2 shows high magnification of CD3 + CD4 + co-expression to confirm T cell presence in the tissue.

Collectively, these data show that exogenously added CCL27 protein promotes the relocation of tissue-resident T cells in the foreskin.

## Discussion

It is not only important to understand the mechanisms of HIV acquisition in males, if a more holistic approach to addressing the HIV epidemic can be developed, but also what may constitute risks to becoming HIV infected in males who do not elect to undergo medical male circumcision (MMC). Although MMC significantly reduces HIV infection risk by up to 60% in African men,^1–3^ the mechanisms that support the foreskin as the main portal of HIV entry in the male genital tract are poorly understood. The implementation of MMC in many African countries, including South Africa, calls for us to examine this tissue in detail. Our focus on adolescent males in regions of high HIV prevalence in South Africa, meant we could not only identify risk factors associated with the potential for HIV infection but also gain insight into mechanisms of how MMC reduces viral acquisition. The most common STI in our semi-rural cohort was *Chlamydia trachomatis*, a Gram-negative obligate intracellular bacterium that infects epithelial cells of the urethra and remains asymptomatic in 50% of infected men.^25^ We show in this study that *Chlamydia trachomatis* induces significant changes in the location and density of HIV target cells in both the outer and inner foreskin, which appear to have an established set of chemokine and chemokine-associated gene expression differences. These data imply that MMC efficacy is likely due to the removal of tissue that is rich in HIV targets,^26^ which are increased in numbers in the presence of an urethral STI.

We showed that elevation of CCL27 in the inner foreskin relative to the outer foreskin was the strongest signal and suggests that this chemokine may be important for movement of T cells to the inner foreskin for providing potential protective immunity. In the context of HIV, highly abundant CCL27 may enhance HIV target cell presence, and hence susceptibility to infection. The importance of CCL27 in HIV susceptibility is not clear, but high levels of CCL27 expression are likely to cause an influx of potential HIV target cells expressing CCR10, the receptor for CCL27.^22^ Indeed, skin-homing LCs and T cells have both been shown to express CCR10.^23^ CCR10 expression has been used to identify Th22 cells that are involved in tissue repair and epithelial regeneration.^22^ Given that Th22 cells have high expression of CCR5, studies have suggested that these cells are highly susceptible to HIV infection.^22^ Whether the enhanced CCL27 signal, we identified in the inner foreskin, is responsible for an influx or induction of CCR10-expressing HIV target cells warrants further investigation. Our experiments of adding exogenous CCL27 to inner foreskin tissue cultures did reveal that this chemokine induced the movement of tissue-resident CD3^+^CD4^+^ T cells to the epithelium of the inner foreskin and may be compatible with in vitro monolayer and organotypic skin cultures showing that CCL27, expressed by epidermal keratinocytes,^27^ enhanced the influx of CCR10^+^CD4^+^ T cells into inflamed skin. Our CCL27 data also appear to be reminiscent of studies that have investigated wound healing,^27,28^ where it has been shown that upon burn-wound injury, CCL27 produced by keratinocytes promotes tissue repair. It is possible that the source of CCL27 expression in our study is also from keratinocytes, and may be a result from the MMC wounding procedure itself. Whilst we cannot discount this, the fact that both groups of recruited adolescents (STI- controls vs STI+) were subjected to the same procedures and that laboratory tissue processing was identical, this would tend to favour a pre-existing CCL27 differential signal between the inner and outer foreskin rather than a result of mechanical wounding. We speculate that the constitutive higher CCL27 expression in the inner foreskin allows rapid movement of HIV target cells to this site. The primary site of *Chlamydia trachomatis* infection in the male genital tract is the penile urethra, and specifically the single-cell columnar layer of the urethral epithelium.^25^ *Chlamydia trachomatis* is a chronic persistent infection and induces pro-inflammatory cytokine production by epithelial cells via TLR2 and TLR4 engagement.^29^ Interestingly, we found that TLR2 gene expression was higher in the outer foreskin, and that TLR4 was higher in the inner foreskin, although seemingly unrelated to the presence of *Chlamydia trachomatis*. These data would suggest that the foreskin is pre-disposed to signals leading to inflammation. It has been shown that pro-inflammatory cytokines, IL-1α and TNF can trigger the release of CCL20 in response to skin irritants, but it is unclear what the triggering signals are for CCL27 expression.^30^ A recent report characterised the cytokine profile of the urethra, fossa and glans and showed that CCL27 was expressed at low levels in all three regions.^18^ However, high levels of CCL28, which also binds CCR10, were detected along with increased proportions of T-cells expressing CCR6 and CCR10.^18^ This suggests that the chemokine milieu in penile tissue may favour recruitment of CCR10^+^ immune cells. Interestingly, also in our study, CCL28 gene expression was elevated in the inner foreskin, although to a much lesser degree.

CXCR7 was also elevated in the inner foreskin, except in the presence of an STI, where it was elevated in the outer foreskin. The function of CXCR7 is poorly characterised and unknown in the context of mucosal immunology, but has been proposed to bind to CXCL12 the canonical ligand for CXCR4,^31^ suggesting that the biology of CXCR4 and CXCR7 may somehow be interconnected, as shown in the context of intestinal mucosal homoeostasis and inflammatory bowel disease^32^ In our study, we detected higher expression of CXCL12 and CXCR7 genes in the inner foreskin, and it may be conceivable that high expression of CXCL12 may attract and retain CXCR7^+^ T cells into this site. The observation that CXCR7 was higher in the outer foreskin from individuals with an STI compared with individuals without an STI warrants further investigations, but possibly suggests that CXCR7 may play an important role in the outer foreskin immunity in the presence of an STI.

Previous reports have shown an increased number of HIV target cells in the inner foreskin when compared with the outer foreskin.^16,17^ Whether these differences reflect a steady-state, physiological phenomenon is unclear, but prior studies did not consider the influence of potential STIs on possible HIV target cell infiltration. This is important given that study participants are often at risk of acquiring STIs.^16,33^ Consequently, our findings suggest that STIs are a potential underlying cause for increased recruitment of HIV target cells to the inner foreskin in uncircumcised males. Studies using a milieu of inflammatory signals, perhaps those which might be observed following an STI infection,^34^ showed increased infiltration of CD4^+^ T cells and LCs into the inner, but not the outer, foreskin following stimulation with TNF-α.^35^ We identified three main findings from our study about the location of CD4^+^ cells and LCs that may be instrumental to understanding HIV acquisition and, by inference, the protective mechanism of MMC. First, that the density of the dual-expressing CD4^+^CCR5^+^ cells was enriched in the epithelium of both the outer and inner foreskin in the presence of STIs; second, that > 90% of CD4^+^ T cells co-expressed CCR5 in the epithelium, being in agreement with Prodger et al.^15^ who showed that CD4^+^CCR5^+^ cells were more than fourfold abundant in the foreskin tissue relative to the peripheral circulation. Third, that LCs were closer to the keratin layer in the outer foreskin relative to the inner foreskin. The finding that HIV target cells are close to the lumen and are increased in density within this locale when infected with an asymptomatic STI, provides tantalising clues to the role of proximity and spatial arrangement of these cells in HIV acquisition in males with intact foreskin.

The foreskin provides barrier integrity and immune surveillance to the continuous bombardment of sexually acquired infectious and non-infectious agents, and is likely to be in a state of inflammation.^5,16^ An important component of barrier integrity is the keratin layer, and we explored whether there were differences in keratin thickness upon an STI infection. Although studies have shown no biological differences in keratin thickness between the outer and inner foreskin, and hence no relation to the mechanism of reduced HIV acquisition after MMC,^11,12^ the impact of STIs on epithelial keratinisation has not previously been addressed. We show that despite a higher mean (2.67 µm) keratin thickness in the outer foreskin relative to the inner foreskin, the presence of an asymptomatic STI did not associate with lower epithelial keratinisation in either tissue. It is possible, however, that differences in barrier function may exist between the outer and inner foreskin, as previously reported.^35^ Lower barrier integrity could be related to enhanced inflammation and loosening of tight junctions^21^ and the presence of pro-inflammatory T cells.^25^ How this might promote HIV acquisition has yet to be determined.

This is the first study to show that asymptomatic STIs, particularly *Chlamydia trachomatis* infection, are associated with altering HIV target cell density in the foreskin. Could it be that pre-existing chemokine gradients across the outer and inner foreskin would allow an uncomplicated passage of CD4^+^ T cells to either the outer or inner foreskin? When we tested the hypothesis that CCL27 would result in migration of T cells to the inner foreskin, we identified that exogenously added CCL27 resulted in relocation of tissue-resident CD3 + CD4 + T cells into the epithelium. These data provide direct evidence for the ability of CCL27 to cause T-cell migration in the foreskin and ascribes a mechanism to the differential CCL27 gene signal we observed between the outer and inner foreskin in tandem with elevated CD4 + T cells in the epithelium.

Our study has certain limitations, one being that the study sample size was small, particularly for STI positive participants. However, the main feature from our data is that a predominantly urethral infection of *Chlamydia trachomatis* can impact on immune events in the foreskin. Much of the data generated in this study was done so in a blinded manner, involving three international laboratories and was rigorously analysed for statistical significance, including corrections for multiple comparisons. Hence, the data generated provides credibility to the limited numbers of samples.

In conclusion, our data show four key findings: (1) there are constitutive differences in chemokines between outer and inner foreskin tissue; (2) that a urethral STI is able to induce inflammation in a distal location of the foreskin, and that these changes are found in both the outer and inner foreskin; (3) the inner foreskin may be more susceptible to HIV infection due to increased proximity of HIV target cells to the surface of the tissue and (4) this increased proximity may be due to differences in CCL27 expression between the outer and inner foreskin. That HIV target cells are located in defined anatomical areas in both the outer and inner foreskin suggest a protective mechanism of MMC.

## Materials and methods

### Study participants

MMC is offered as a free service to adolescents and adults as a standard of prevention care against HIV and other STIs in South Africa. From February 2013 to October 2014, we recruited 109 HIV uninfected males (aged between 13 and 24 years) from a semi-rural community in KwaZulu-Natal (Edendale). An additional 41 males (13–24 years) were recruited from an urbanised community in Soweto. Study participants provided written informed consent and, in the case of minors, parental assent was granted for the collection and analysis of foreskin tissues that would otherwise be discarded. Physical exams were conducted to determine evidence of pre-existing STIs. Men with symptomatic STIs were ineligible and referred for treatment.

### Sample collection and processing

Circumcision was performed using the dorsal slit method either with or without the aid of a clamp and foreskins were shipped within 24 h to the laboratories in Cape Town for processing. Tissues were separated into inner and outer foreskins. Smaller, 5–7 mm^2^-sized sections were excised and snap-frozen in self-made foil cryomolds containing optimal cutting temperature (OCT) freezing medium (Leica Biosystems, Nussloch, Germany). Tissue samples used for the chemokine gene array were stored and frozen in RPMI with 10% dimethyl sulfoxide (DMSO). In addition, first-pass urine and penile swabs were collected for STI analysis.

### Blinding of samples

Samples were analysed in a blinded fashion for CD4^+^CCR5^+^ cells, chemokine gene expression and CCL27 protein expression. OCT-mounted tissues were sent blinded to Northwestern University: 21 sets of inner and outer foreskin blocks from STI + and 21 from age-matched STI- participants were sent for fluorescent imaging. Likewise, 28 sets of inner and outer foreskin tissue were sent blinded to the Karolinska Institutet for the 84 targeted gene array. This consisted of 18 sets of inner and outer foreskin blocks from STI + and 10 from age-matched STI participants. Only after the data were generated were the samples unblinded and then analysed to identify differences between inner and outer foreskin and between STI + and STI- age-matched differences.

### Detection of STIs

Two real-time multiplex PCR (M-PCR) assays were used to detect *Neisseria gonorrhoeae* (NG), *Mycoplasma genitalium* (MG), *Trichomonas vaginalis* (TV), *Chlamydia trachomatis* (CT) and Herpes Simplex Virus Types 1 and 2 (HSV-1 and 2), as previously described.^36^ DNA extraction was performed using the X-tractor gene platform (Qiagen, Germany), and M-PCRs were performed using Rotor Gene 3000 and 6000 platform (Corbett Research, Australia).

### Chemokine and chemokine receptor gene array

Frozen inner and outer foreskins were defrosted, and washed three times in sterile PBS in order to remove any trace of DMSO. The tissues were placed in a microcentrifuge tube containing cell lysis buffer (RLT buffer, RNeasy Mini kit, Qiagen) and cut into small pieces. The tissues were then homogenised on ice using an electronic dispersion unit from VWR model VDI 12 (VWR International AB, Stockholm, Sweden) or a TissueRuptor (Qiagen, Dusseldorf, Germany) in two 5 s intervals. The homogenised sample was centrifuged in a benchtop microfuge at 10,000×*g*, and the supernatant was collected. RNA was extracted using the RNeasy Mini kit (Qiagen, Qiagen Nordic, Sweden) according to the manufacturer’s instructions. The quality and quantity of the isolated RNA were determined using the NanoDrop 2000c (Thermo Scientific, MA, USA). Equal amounts of isolated RNA (2 μg/sample) was used for preparation of the cDNA using the RT^2^ first strand kit (Qiagen), according to the manufacturer’s instructions. The human chemokine and chemokine receptor PCR array (PAHS-022ZA, Qiagen), containing 84 genes of the CC and CXC motif subfamilies and other related genes, was used. The plates contained five housekeeping genes (ACTB, B2M, GAPDH, HPRT1 and RPLPO) to normalise array data. cDNA prepared was mixed with the PCR master mix, RT^2^ SYBR green ROX (Qiagen), and pipetted on to the plate according to the manufacturer’s instructions. Plates were analysed on a TaqMan QuantStudio 7 flex system (Applied Biosystems, Life Technologies, NY, USA), and data were obtained as Ct (cycle threshold) values.

### Immunofluorescent staining of HIV target cells and confocal microscopy

Cryosections of ~8–10 μm were sectioned from matching frozen inner and outer foreskin OCT blocks and transferred onto glass slides. Tissue sections were fixed with 3.7% paraformaldehyde (Santa Cruz Biotechnology) for 10 min at room temperature. Sections were blocked with 2% bovine serum albumin for 30 min at room temperature. Monoclonal mouse anti-human α-filaggrin (Santa Cruz Biotechnologies), CD207 (Beckman Coulter), CD4 (Sigma-Aldrich) and monoclonal rabbit anti-human CD3 (Abcam) were used as primary antibodies. In addition, a hybridoma-raised monoclonal mouse anti-human CCR5 was used as a primary antibody (obtained from Mathias Mack, University of Regensburg, Germany). Rhodamine red-conjugated donkey anti-mouse (Jackson ImmunoResearch), Cy3-conjugated and Cy5-conjugated donkey anti-mouse (Amersham Biosciences), Alexa Fluor 594-conjugated and 488-conjugated goat anti-rabbit antibodies were used as secondary antibodies for staining. Slides were washed in 1× PBS in between fixation, blocking and antibody incubations. Counterstaining with Hoechst 33342 (Life Technologies) allowed visualisation of nuclei. Imaging was performed using a Zeiss LSM 510 M Confocal Microscope (Zeiss, Germany). For each tissue section, a minimum of five images spanning the section (stitched panels), were captured at either 40 × (Filaggrin, CD207) or 60 × (CD4) magnification. Maximum intensity projections of stitched panels were used for analysis using an interactive programme designed using the programming language IDL (Interactive Data Language, Harris Geospatial Solutions, Inc.), as described previously.^12^ Filaggrin thickness was quantified from a total of five images per inner and outer foreskin from age-matched STI− and STI + males. Simultaneous imaging of CD4 and CCR5 (Fig. 3, S1) and quantitative analysis of CCL27 (Fig. 5) was conducted utilising a DeltaVision Elite System and SoftWorx software (GE Biosciences). For each section of tissue analysed, at least ten images were captured.

### Flow-cytometric analysis of foreskin epidermal T cells

Fresh inner and outer foreskin tissue were processed within 2–4 h following circumcision. Both inner and outer foreskin tissues were dissected identically, but separately, into ~1 × 1 cm^2^ pieces and migratory immune cells harvested, as previously described for human skin samples.^37^ Briefly, dissected foreskin samples were incubated in dispase enzyme solution (HBSS supplemented with 5 mg/mL dispase enzyme; Gibco Life Technologies) for 18 h at 4 °C. The following day, the epidermis was separated from the underlying dermal layer using forceps. The epidermal sheets were cultured in complete medium (RPMI-1640 supplemented with 10% FCS, 100 U of penicillin/ml and 100 μg of streptomycin/ml) for 48 h to allow cells to spontaneously migrate from the epidermis into the surrounding culture media. After migration, the cells were harvested and counted. Approximately, 2 × 10^6^ cells were stained with fluorophore-conjugated antibodies against CD3 (UCHTI), CD4 (SK3) and CCR5 (J418F1) for 20 min at room temperature and analysed on an LSRII flow cytometry system (BD). All antibodies were purchased from Biolegend. The data were analysed using FlowJo version 10 (FlowJo, LLC).

### Stimulation of inner foreskin tissue with TNF and CCL27

Inner and outer foreskin tissues were collected from 13 participants undergoing MMC and dissected into roughly equally sized explants (of 0.5 × 0.5 cm) and cultured in complete medium (RPMI-1640 supplemented with 10% FCS, 100 U of penicillin/ml and 100 μg of streptomycin/ml) with either nil (unstimulated), TNF (100 ng/mL) and CCL27 (400 ng/mL) for 48 h at 37 °C, 5% CO_2_. Tissue was then placed into regular cryomolds, embedded in optimal cutting temperature (OCT) solution and frozen at −80 °C. Cryosections of ~12 μm were sectioned and fixed in 3.7% formaldehyde in PIPES buffer (2 mM MgCl_2_/1 mM EGTA/100 mM PIPES pH 6.8) for 10 min at room temperature. The tissues were washed three times in PBS and incubated in blocking solution (10% normal donkey serum, 0.1% Triton X-100, 0.01% NaN_3_) for 1 h at room temperature. The tissue sections were incubated with primary antibodies against human CD3 (Abcam; neat) and CD4 (Sigma-Aldrich; 1:200) for 1 h at 37 °C. Following this, the sections were incubated for 30 min at room temperature with the respective species-specific secondary antibodies: donkey anti-mouse Cy5 (1:500) and donkey anti-rabbit Alexa Fluor 488 (1:500). Tissue sections were counterstained with DAPI (0.4 µg/ml) for 5 min at room temperature, and mounted using fluorescent mounting medium (Dako). Between all antibody and counterstaining steps, slides were washed in PBS three times. All experiments were set up in a blinded manner, with the laboratory staff not knowing which was inner, outer or the stimulation conditions.

### Imaging and image analysis

All images were visualised in a blinded manner, and obtained by deconvolution microscopy on a DeltaVision RT system collected on a digital camera (CoolSNAP HQ; Photometrics) using a ×60 or ×100 oil objective. To determine target cell density in foreskin samples, we took ten measurements per tissue block for each tissue stimulation condition. Panel images were acquired to include the epithelium and lamina propria. Each image consisted of a stitched panel comprised three ×60 images across the lumen and *n* ×60 images to the basal layer, *n* being dependent on the epidermal thickness of each sample. These images were collected at the University of Cape Town and uploaded to the cloud for analysing at Northwestern University in a blinded manner. Using the SoftWorX software, all CD3 + and CD4 + cells were separately circled and the area of the epithelium was measured. Following, for each image, circled CD3 + and CD4 + cells were analysed using IDL and specifically created algorithms in order to obtain a count of double-positive intraepithelial cells. Finally, the number of CD3 + CD4 + target cells in each sample was divided by the average epithelial area in to calculate density. Once this was ascertained, the data were unblinded and analysed.

### Data analysis

Statistical analyses were performed using R version 3.5.0, GraphPad Prism version 6.0® (GraphPad Software, San Diego, CA, USA), and STATA^TM^ version 11 (StataCorp, TX, USA). To perform group comparisons, different generalised linear mixed-effects models were fitted using the best fitting model, depending on the nature of the data analysed. We always included donor as a random effect in the models. Keratin thickness was modelled using a log-normal model, all cell count data were modelled with a negative binomial model, using the area analysed in each image as an offset, and the cell distance from the outer keratin layer was also modelled by a log-normal model. All possible contrasts within each model were performed, correcting for multiple comparisons using Bonferroni method. A *q*-value < 0.05 was always used as significance cut-off. For gene array data, we used the empirical Bayes moderated linear models implemented in the limma package^38^ with the ΔCT values obtained after normalisation with the Housekeeping genes included in the array. We estimated the effects of STI (including all STIs), sample type and the combination of these two factors, controlled for within participant correlation, and tested for differential expression in all the possible contrasts within the model, using Benjamini Hochberg (BH) method for multiple testing correction. FDR for differential gene expression was set at FDR < 0.05. In addition, we used PCA analysis and unsupervised Euclidian hierarchical clustering using the Ward’s minimum variance method to visualise the variation in the mRNA levels of the significantly differentially expressed genes of the chemokine array. For the stimulation assay, we fitted a negative binomial generalised linear mixed-effects model to the cell counts per image including the stimulation used in the model, controlling for within donor correlation, and using the area analysed per image as an offset of the model. We performed pairwise comparisons between every stimulation group and corrected the *P*-values using Bonferroni to account for multiple comparisons with a cut-off for significance of *q*-value < 0.05.

## Supplementary information

Supplementary Table 1
