# Supplementary material for: Impact of chemokine C–C ligand 27, foreskin anatomy and sexually transmitted infections on HIV-1 target cell availability in adolescent South African males
Source: Mucosal Immunol. 2019 Oct 16;13(1):118–27. doi: 10.1038/s41385-019-0209-6 (PMC6914668; doi:10.1038/s41385-019-0209-6)

Supplementary Table 1: Linear model gene expression, log_2 fold change between the inner and outer foreskin; showing unadjusted and adjusted significance differences.

Comparison	Gene	Fold change	P.Value	adj.P.Val
Inner vs Outer	CCL27	6,94	4,71E-12	3,86E-10
	CXCL12	2,35	5,46E-08	1,74E-06
	TLR4	2,17	6,37E-08	1,74E-06
	SLIT2	1,98	2,07E-07	4,25E-06
	CXCR7	1,63	6,92E-07	1,14E-05
	CCL28	1,67	2,47E-06	3,37E-05
	CCL18	3,05	5,28E-06	6,18E-05
	DARC	1,67	6,50E-06	6,42E-05
	XCR1	2,13	7,05E-06	6,42E-05
	CMKLR1	1,79	9,53E-06	7,82E-05
	IL16	1,96	1,88E-05	1,40E-04
	CMTM4	1,49	2,44E-05	1,56E-04
	GPR17	3,98	2,48E-05	1,56E-04
	PPBP	12,10	3,43E-05	2,01E-04
	CXCR6	1,70	5,01E-05	2,74E-04
	CMTM3	1,30	5,36E-05	2,75E-04
	TYMP	-1,83	7,22E-05	3,48E-04
	CCL13	2,22	1,15E-04	5,22E-04
	CCL17	2,50	3,06E-04	1,32E-03
	TLR2	-1,22	6,60E-04	2,71E-03
	CXCL10	-2,96	1,02E-03	4,00E-03
	CCL21	1,55	1,19E-03	4,43E-03
	CMTM1	1,23	1,52E-03	5,42E-03
	C5	1,05	1,70E-03	5,80E-03
	CCL23	1,72	2,34E-03	7,68E-03
	CCL11	10,97	3,35E-03	1,06E-02
	CKLF	0,78	3,97E-03	1,21E-02
	CCL2	1,19	4,65E-03	1,36E-02
	CCL14	1,98	6,57E-03	1,86E-02
	CCR2	0,96	1,68E-02	4,60E-02
	CCL26	0,88	1,77E-02	4,69E-02
STI Positive vs Negative	NONE			
STI Positive vs Negative in Outer	CXCR7	1,01	2,49E-04	2,04E-02
STI Positive vs Negative in Inner	NONE			
Inner vs Outer in STI Negative	CCL27	3,36	1,87E-06	1,53E-04
	CXCR7	1,12	1,28E-05	5,25E-04
	TLR4	1,19	8,20E-05	2,24E-03
	DARC	1,10	1,58E-04	3,23E-03
	GPR17	2,61	4,28E-04	6,54E-03
	SLIT2	0,99	4,78E-04	6,54E-03
	CCL18	1,63	1,43E-03	1,45E-02
	CXCL12	1,00	1,48E-03	1,45E-02
	CCL28	0,85	1,62E-03	1,45E-02
	PPBP	7,10	1,77E-03	1,45E-02
	CKLF	0,68	2,12E-03	1,58E-02
	TYMP	-1,08	2,64E-03	1,80E-02
	CXCR6	0,95	3,28E-03	2,07E-02
	TLR2	-0,79	5,36E-03	3,14E-02
	CMKLR1	0,84	6,00E-03	3,28E-02
	CCL16	4,04	8,36E-03	4,28E-02
Inner vs Outer in STI Positive	NONE*			
	*CXCR7	-1,74	9,12E-04	7,48E-02

Supplementary Figure 1

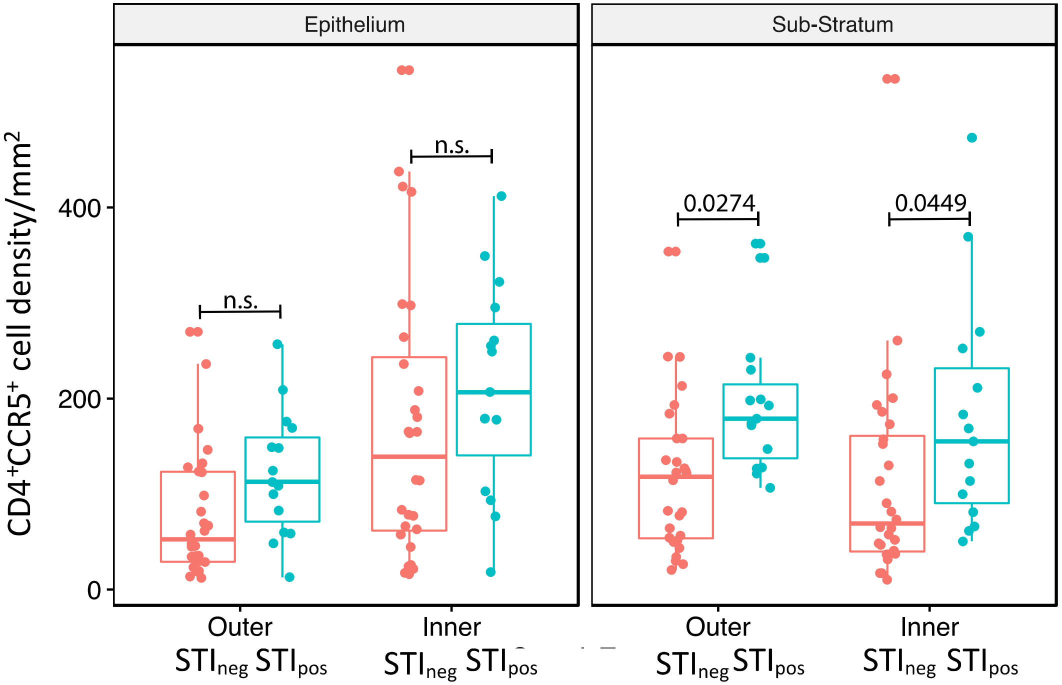

Supplementary Figure 2

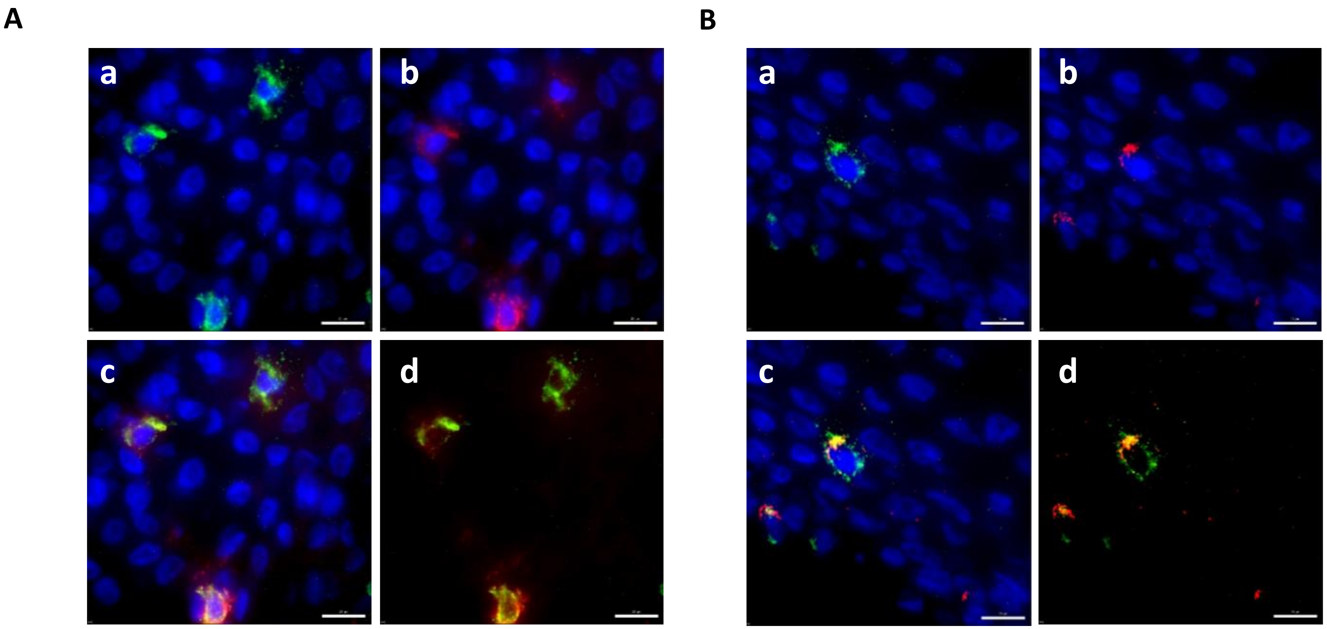

Supplementary Figure 3

A

CD207
Isotype
Control

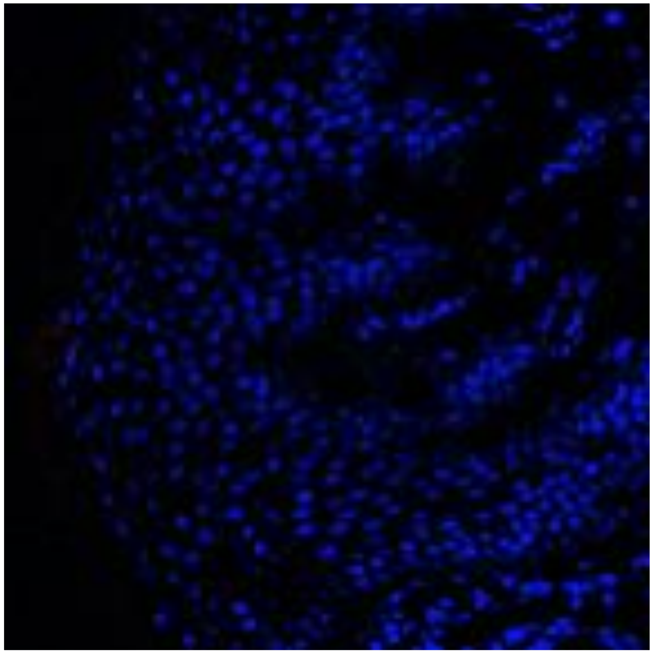

Supplement: Supplementary file 1 — Supplementary Table 1 [file 41385_2019_209_MOESM1_ESM.pdf]
